# Preparation and characterization of a new Gd_2_O_3_-epoxy composite for neutron shielding applications

**DOI:** 10.1038/s41598-024-77070-w

**Published:** 2024-10-27

**Authors:** Seyed Mohammadreza Safavi, Mohammad Outokesh, Naser Vosoughi, Amin Yahyazadeh, Aghil Mohammadi, Mohammad Amin Kiani, Seyed Sajad Jabalamelian

**Affiliations:** 1 Department of Energy Engineering, Sharif University of Technology, Azadi Ave., P.O. Box: 11115-1639, Tehran, Iran; 2 Department of Energy Engineering and Physics, Amirkabir University of Technology, Enghelab Ave., P.O. Box: 15875-4413, Tehran, Iran

**Keywords:** Neutron shields, Epoxy composite, Gadolinium oxide, Monte Carlo simulation, Mechanical strength, Chemistry, Engineering, Materials science, Physics

## Abstract

The current study aims to introduce a new polymeric composite consisting of epoxy resin as the matrix and gadolinium oxide (Gd_2_O_3_) as the neutron adsorption ingredient. The shielding performance of the composite was assessed by neutron attenuation experiments with an Am-Be source and polyethylene moderator. The results of these experiments showed an appreciable agreement with the Monte Carlo simulations. Other characteristics of the composite, including mechanical strength, thermal stability, microtexture, and its chemical compositions, were examined using standard tensile test, thermogravimetric analysis, X-ray diffraction, scanning electron microscopy, static light scattering analyses, and Fourier-transform infrared spectroscopy (FTIR). The results indicated that the new composites offer appreciable neutron absorption properties so that samples with 0.5%, 2%, 5%, and 10% Gd_2_O_3_ content could reduce the neutron beam intensity by 54%, 63%, 66%, and 70% at a thickness of 4 cm.

## Introduction

Widespread applications of radioisotopes and other sources of elementary particles, e.g., accelerators and reactors, have given rise to significant risks of exposure to ionizing radiation^1–4^. One of the most hazardous ionizing radiation is neutron, whose great deal of usage in medicine (e.g., neutron capture therapy)^5,6^ , industry (e.g., nuclear power plants, online elemental analyses)^7,8^, and science (neutron microscopy and imaging) has necessitated the effort for the development of effective neutron shields^9^.

Concrete has been traditionally used as the most exploited neutron shield^10^ despite its apparent deficiencies, such as transportation problems and its heavy and inflexible nature^11,12^. Recently, however, attention has been drawn more toward polymeric composites due to their light weight, mechanical strength, corrosion resistance, and easier fabrication processes^13,14^.

As the matrix of neutron shields, various polymers have been utilized from both thermoset and thermoplastic groups^15^. Polymeric matrices that comprise hydrogen, oxygen, and carbon provide a large cross-section for the scattering of fast neutrons and reducing their energies into the thermal energy range^16^. The thermal neutrons, then, are captured by the absorber atoms, which are incorporated into the composite structure^17–19^. This combined effect makes the polymeric composites efficient neutron shields^20^.

One of the most applied thermoplastic polymers in neutron shielding is polyethylene, in both of its high—density (HDPE) and low-density (LDPE)forms. Shang^21^ fabricated a multilayer neutron shield consisting of alternating HDPE/Hexagonal boron nitride (h-BN) and LDPE layers through a two-step hot-pressing process. In this composite, the hexagonal boron nitride (h-BN) in the HDPE/h-BN layers is highly oriented in the in-plane direction. The composite maintains a stable, continuous multilayer structure with strong adhesion between the adjacent layers. This PE/h-BN composite demonstrates effective neutron shielding capabilities. Specifically, when the filler content is 30 wt%, the multilayer PE/h-BN film with a thickness of 70 $\mu {\text{m}}$ exhibits a $\frac{I}{{I_{0} }}$ value of 4.16%. In addition to PE, other thermoplastic polymers such as polystyrene, polyurethane, and silicon have also been used in recent works. Vegari et al.^22^ investigated boron carbide and boric acid nanoparticles as thermal neutron absorbents, using high-density polyethylene (HDPE) as a neutron moderator. The aim was to assess the performance of those particles as the nanoshields against photo neutrons generated by the medical linear accelerators. The nanoshield comprising 5% boron carbide and 10% boric acid, with a macroscopic cross-section of 0.960, demonstrated the best performance. Hassanpour et al.^23^ introduced two new composites: Polymer/h-BN and Polymer/B_4_C, and assessed their neutron absorption abilities in a medical accelerator facility. Simulations showed that the Polymer/h-BN layer reduces neutron leakage by up to 60.43% and in bunker walls by 95.61%. The PL/B_4_C layer at the same conditions achieved 44.32%, 80.46%, and 70.59% reduction rates. The PL/h-BN layer outperformed the PL/B_4_C layer in reducing neutron leakage and photon flux, demonstrating its superior shielding effectiveness. Thus, this investigation highlights the wide-ranging applications of neutron-polymeric shields in medical fields. Ghassoun et al.^24^ used the MCNP5 code to simulate a radiotherapy room with an 18 MV medical linear accelerator to evaluate neutron and secondary gamma-ray fluences, energy spectra, and dose distributions within a tissue-equivalent phantom. The simulation revealed that paraffin wax containing boron carbide effectively reduces both neutron and secondary gamma doses. For instance, a 2.5 cm Wax/B_4_C shield can reduce the total dose by approximately 4.12% on the surface of a tissue phantom.

Meanwhile, thermoset polymers such as epoxy resins also have been used in the fabrication of a wide range of neutron shields. The appreciable mechanical and thermal stability of epoxy resins, as well as the simplicity of their application, arising from their initial liquid form, makes the epoxy prime matrix of the neutron shields^25^. Okuno, in 2005^26^, showed that the shielding performance and mechanical strength of epoxy shields are superior to concrete and polyethylene.

The composites used for the neutron shielding must contain elements with a large neutronic absorption cross-section. According to Table 1, Gadolinium, Boron, Samarium, and Cadmium, or a combination of these elements, are the active ingredients of the neutron shields and were used in the previous investigations^27^.

**Table 1:** Thermal neutron absorption of common elements (isotope).

Isotope	Isotopic abundance (%)	0.025 eV Neutron absorption cross section (barn) [JANISWeb]
Boron-10	19.8	~ 3.840
Samarium-149	13.8	~ 40.550
Gadolinium-155	14.8	~ 61.050
Gadolinium-157	15.7	~ 254.250
Cadmium-113	12.2	~ 20.600

Adeli et al.^28^ produced and examined a composite shielding material that used epoxy as the matrix and boron carbide (B_4_C) as a filler to absorb thermal neutrons. His 9.8 mm thick fabricated shield reduced incident neutrons by up to 80% by adding 3% B_4_C. Furthermore, he significantly improved the composite’s shielding performance by more than 60% by incorporating aluminum Tri hydroxide and tungsten trioxide (WO_3_) powder. WO_3_ has been utilized in various types of gamma shields, and as a result, it was integrated into the epoxy/B_4_C composite. Das et al.^29^ also studied a new lead-free full shield of poly methyl methacrylate (PMMA) and tungsten (W) to reduce the total dose of coupled neutron-gamma radiation fields using FLUKA simulations. They concluded that only 25% of W can decrease the gamma dose to a very low level. Additionally, they optimized the shield and found that a 70% W is the optimal composition against neutron-gamma radiation. In another study, Kiani et al.^30^ designed and reinforced an epoxy/B_4_C composite shield by adding nano clay. They showed that the stability of the shield is appreciably enhanced by this method. According to them, the optimal concentration of nano clay was 3 percent. The data indicated that a macroscopic absorption cross-section of 1.047 cm^−1^ could be achieved by adding 20 percent B_4_C. Regarding the Gd_2_O_3_-containing composites, Irim et al.^31^ designed a neutron shield that comprised a polyethylene matrix and investigated its neutron attenuation performance by adding different concentrations of h-BN and Gd_2_O_3_. They achieved the best absorption efficiency by adding 3% Gd_2_O_3_ and 11% h-BN. In a computational approach, Castley et al.^32^ investigated the shielding performance of a silicone rubber composite that contained three different neutron adsorbers, namely Gd_2_O_3_, Sm_2_O_3_, and B_4_C. It was shown that when thoroughly mixed, a composite containing 10% Gd_2_O_3_ and 2% B_4_C can exhibit the most effective neutron attenuation while maintaining a lower photon radiation dose compared to the 5% borated polyethylene material. As mentioned before, samarium is also a thermal neutron absorber. Toyen et al.^33^ dispersed samarium oxide in an ultra-high-molecular-weight polyethylene shield and applied samarium oxide at 10, 20, 30, 40, and 50 percent concentrations. According to these authors, the optimal concentration of Sm_2_O_3_ was somewhere between 10 and 20%.

Among the stable elements of the periodic table, gadolinium possesses the highest absorption cross-section for the thermal neutrons. The current study aimed to fabricate a new neutron-shielding composite, through compounding the epoxy resin as the polymeric matrix and gadolinium oxide as the neutron absorber. Fast neutrons typically lose energy through elastic collisions with light atoms of polymers, and the likelihood of their absorption by the surrounding heavy material increases. We chose gadolinium oxide as the thermal neutron absorber because its appreciable neutron cross-section allows its application in low concentrations, with an effectiveness comparable to high concentrations of other absorbers. After the fabrication of the composite, it was characterized by different physicochemical methods to disclose its chemical, structural, mechanical, and thermal properties. The neutron attenuation behavior of the sample was examined using an Am-Be source. Attention was also drawn to the simulation of the neurotic response of the fabricated samples using the Monte Carlo method. This was done to achieve a deeper insight into the neutronic behaviors of the manufactured composite.

## Experimental studies

### Materials

The epoxy resin used in the current study was based on bisphenol A, and its hardener was a polyamine. These materials, with the respective trademarks of ML-506 and HA-11, were purchased from the Mokarrar industrial company, Tehran, Iran. The viscosity and density of the ML-506 resin at 25 °C were 1450 cP and 1.11 g/cm^3^, respectively. The resin had an aliphatic structure that increased the flexibility of the produced composite.

Reagent-grade gadolinium oxide powder (Gd_2_O_3_) of 99.9% purity was purchased from Sigma-Aldrich.

### Composite fabrication

According to the manufacturer’s instructions, the weight ratio of resin to hardener was taken 100:15. Fabrication of composite was started by pouring adequate amounts of resin and hardener into a laboratory beaker and their rigorous mixing, using a stirring agitator, which rotated at 400 to 450 rpm, for 15 min. At this stage, gadolinium oxide powder was gradually added to the resin mixture, and the rotation speed of the agitator was increased to 650 to 700 rpm for an additional 45 min. Afterward, the homogenized mixture was transferred to Petri dishes whose diameter was 5 cm, and its temperature was controlled at room temperature for 5 to 6 h, by the air stream of a blowing fan. The reaction was complete at this point, and the formed composite became dry and hard. Composites with different Gd_2_O_3_ content ranging from 0.5% to 10%, along with a neat epoxy sample, were fabricated and tested in the current study.

Given the volume of the composite sample as “V,” the required mass of resin, hardener, and gadolinium oxide were calculated by the following formula:

1 $$m_{composite} = \frac{V}{{\left[ {\frac{x}{{\rho_{Gd2O3} }} + \frac{1 - x}{{\rho_{Epoxy} }}} \right]}}$$

2 $$m_{Resin} = \frac{100}{{115}}m_{Epoxy }$$

3 $$m_{Hardener} = \frac{15}{{115}}m_{Epoxy }$$

4 $$m_{Gd2O3} = \frac{x}{1 - x}m_{Epoxy}$$

where “*x*” denotes mass fraction of Gd_2_O_3_ in the composite, and “${\rho }_{Gd2O3}=7.41 \text{g}/{\text{cm}}^{3}$” and “${\rho }_{Epoxy}=1.11 \text{g}/{\text{cm}}^{3}$” were densities of gadolinium oxide and cured epoxy resin, respectively. The table of fabricated samples, densities, and their composition are shown in Table 2.

**Table 2:** Density and chemical composition of the samples.

Sample name	Density (g/cm^3^)	C (wt%)	H (wt%)	O (wt%)	Gd (wt%)
Neat epoxy	1.16	74.09	7.11	18.8	0
Epoxy/Gd_2_O_3_ 0.5%	1.19	73.72	7.07	18.77	0.43
Epoxy/Gd_2_O_3_ 2%	1.29	72.61	6.96	18.69	1.74
Epoxy/Gd_2_O_3_ 5%	1.47	70.39	6.75	18.52	4.34
Epoxy/Gd_2_O_3_ 10%	1.79	66.68	6.4	18.24	8.68

### Physicochemical characterization

Static light scattering analysis (SLS, FRITSCH, ANALYSETTE 22, Germany) was used to analyze the particle size distribution of the Gd_2_O_3_ absorber.

Crystalline phases of the fabricated composites and gadolinium oxide were detected by X-ray diffractometry (XRD, X’Pert PRO MPD, Panalytical, Netherlands).

Field emission scanning electron microscopy (FE-SEM, TeScan-Mira III, Czech Republic) was used to study the dispersion states of the absorber particles within the composite matrix, as well as the fracture pattern of the samples under the tensile forces.

Meanwhile, the molecular structure of the composites was studied using Fourier transform infrared spectroscopy (FTIR, Thermo, AVATAR, USA).

Another significant property of the composite was its mechanical strength, which was evaluated by tensile strength analysis according to ISO standard 6892. The system employed in this experiment was a tensile testing machine model H10KS, Hounsfield, USA.

To check heat resistance and thermal stability of the prepared composites, a thermogravimetric analysis (TGA, Mettler Toledo, Switzerland) was undertaken with a temperature rise of 10 °C/min in the ambient atmosphere.

### Neutron attenuation experiment

**Fig. 1:**
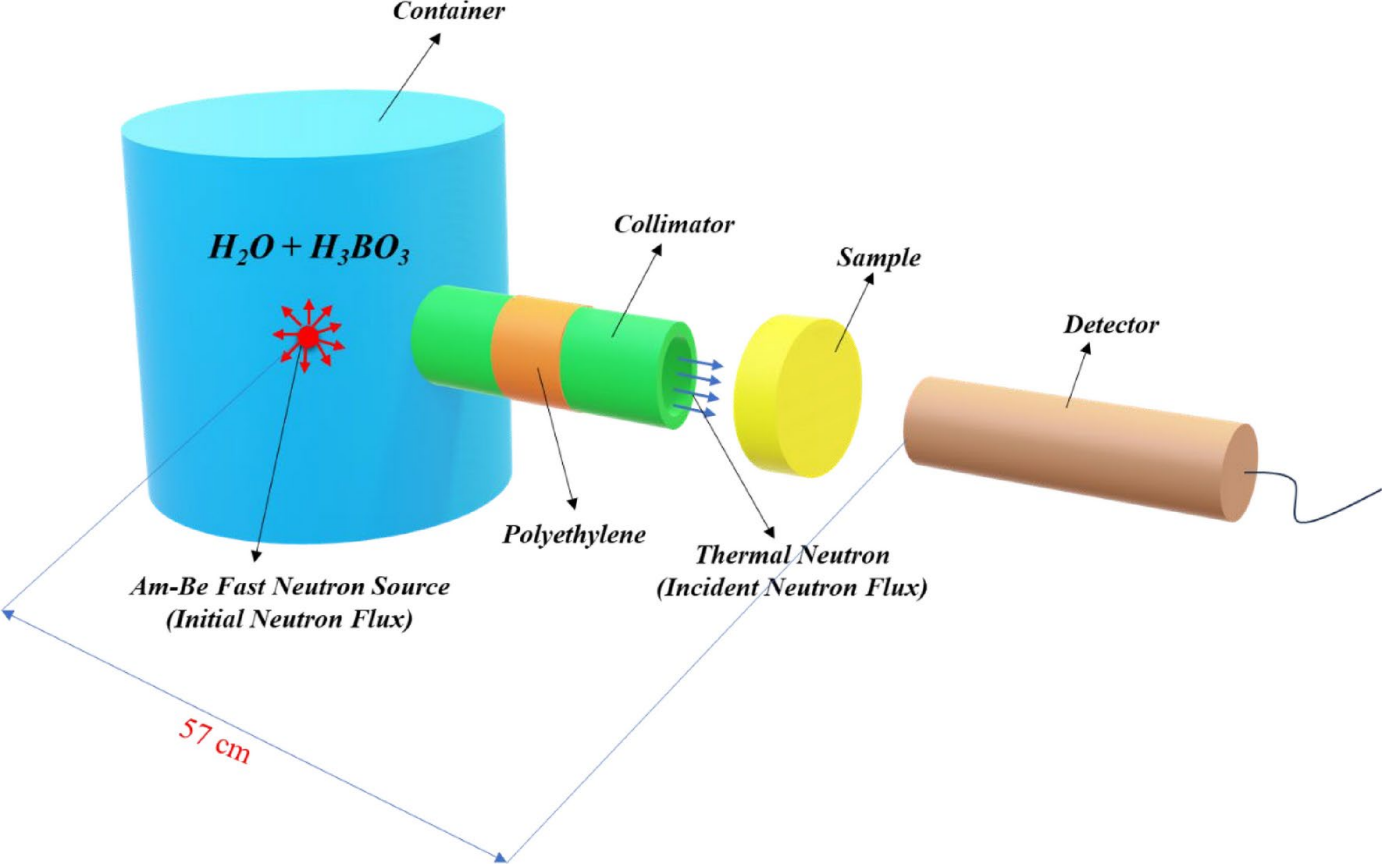
Schematic of neutron attenuation.

The neutron attenuation experiments were carried out using the experimental setup in Fig. 1. According to this figure, an Am-Be neutron source was positioned at the center of a cylindrical container, which was filled with water and boric acid (H_3_BO_3_). Well-collimated thermal neutrons collided with the sample after they were sufficiently thermalized by moving an adequate distance through a polyethylene slab. A ^6^Li glass scintillator, a preamplifier, and an amplifier were used for the neutron detection and counting.

### Monte Carlo simulation

In addition to the experiment, the neutronic response of the fabricated composite was investigated by the Monte Carlo N-Particle (MCNP) computer code, especially its MCNPX version. Our simulation consisted of two steps: At first, the geometry and configuration of the neutronic test apparatus were entered into the MCNP code to result in the intensity and energy spectrum of the incident beam. Thereafter, interactions of the incident beam with the composite specimens, including scattering and neutron capture, were evaluated. In each run of the second step, one million particles were generated and transported through the composite sample.

Given the presence of light materials in the neutron passage and the relatively large dimensions of the equipment, to reduce the computational price, an initial run was conducted to determine the energy and angular distributions of neutron flux at the trailing edge of the collimator. The results indicated that the neutron flux had a predominantly forward angular distribution aligned with the surface’s normal vector. In order to calculate the attenuation coefficient, we utilized the neutron flux output from the collimator and its value at the exit of the shield. Since the interaction rate of thermal neutrons with lithium-6 (resulting in the production of alpha particles and lithium) is measured in a lithium glass detector, this rate can be incorporated into the definition of the attenuation coefficient using the following equation:

5 $$\mu = 1 - \frac{{\varphi_{{{\text{surface}},out}} }}{{\varphi_{{{\text{surface,}}in}} }} = 1 - \frac{{\left( {\Sigma_{n,\alpha } \varphi_{v} } \right)_{{{\text{shield}}}} }}{{\left( {\Sigma_{n,\alpha } \varphi_{v} } \right)_{{{\text{no}} - {\text{shield}}}} }}$$

where $\mu$ is the efficiency of the shield, $\Sigma_{n,\alpha }$ the cross-section of the n-alpha production in the detector, $\varphi$ and the neutron flux. This reaction rate was calculated using the tally F4 and card MT = 205 in the detector cell, with the results presented in the results and discussion section. Considering that the detector’s performance is highly dependent on the energy spectrum of the incident neutron beam, this rate was calculated for 50 logarithmic energy intervals ranging from the thermal region to the fast area. The neutron histories used for the MCNP simulation were 10^7^, and we used pure neutron mode, with a statistical error of less than 0.5%.

## Results and discussion

### Physicochemical characterization

**Fig. 2:**
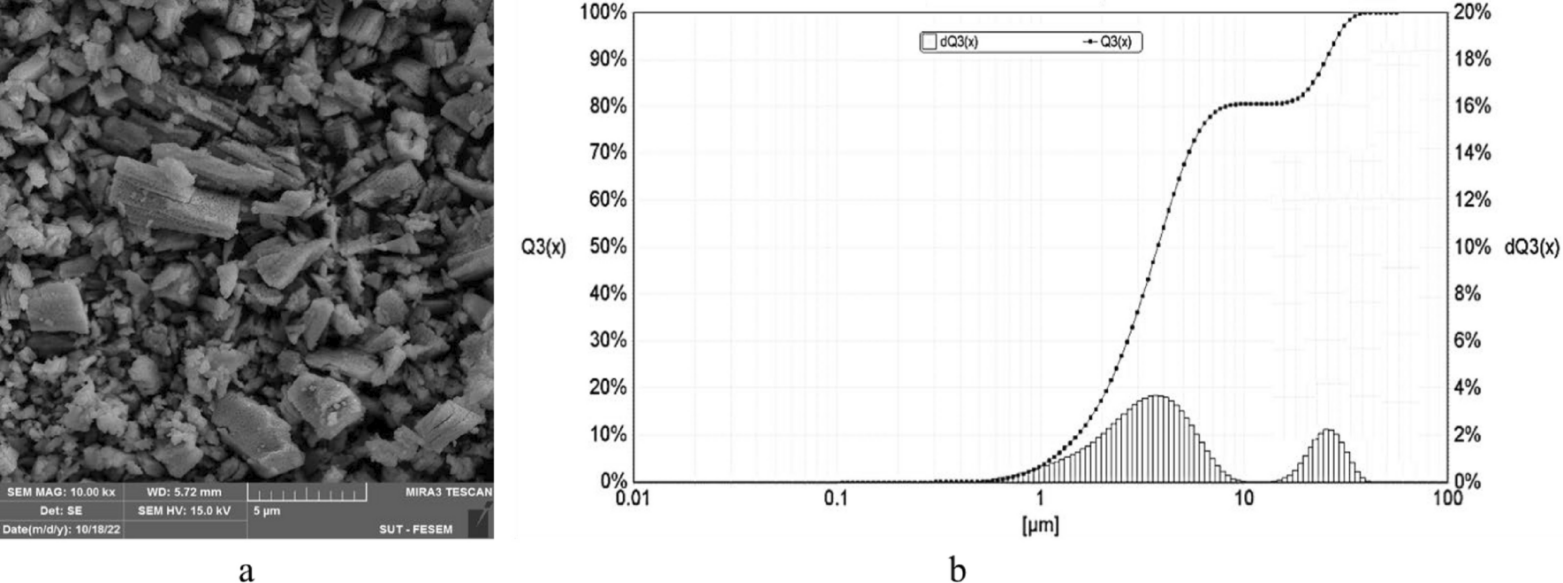
(**a**) SEM image and (**b**) size distribution of Gd_2_O_3_ particles.

The gadolinium oxide particle size distribution was obtained using SLS analysis (Fig. 2b). As it is seen, most of the particles were between 1 and 10 µm. Additionally, the SEM image (Fig. 2a) displays the structure and particle size of Gd_2_O_3_ powder, confirming the SLS results.

**Fig. 3:**
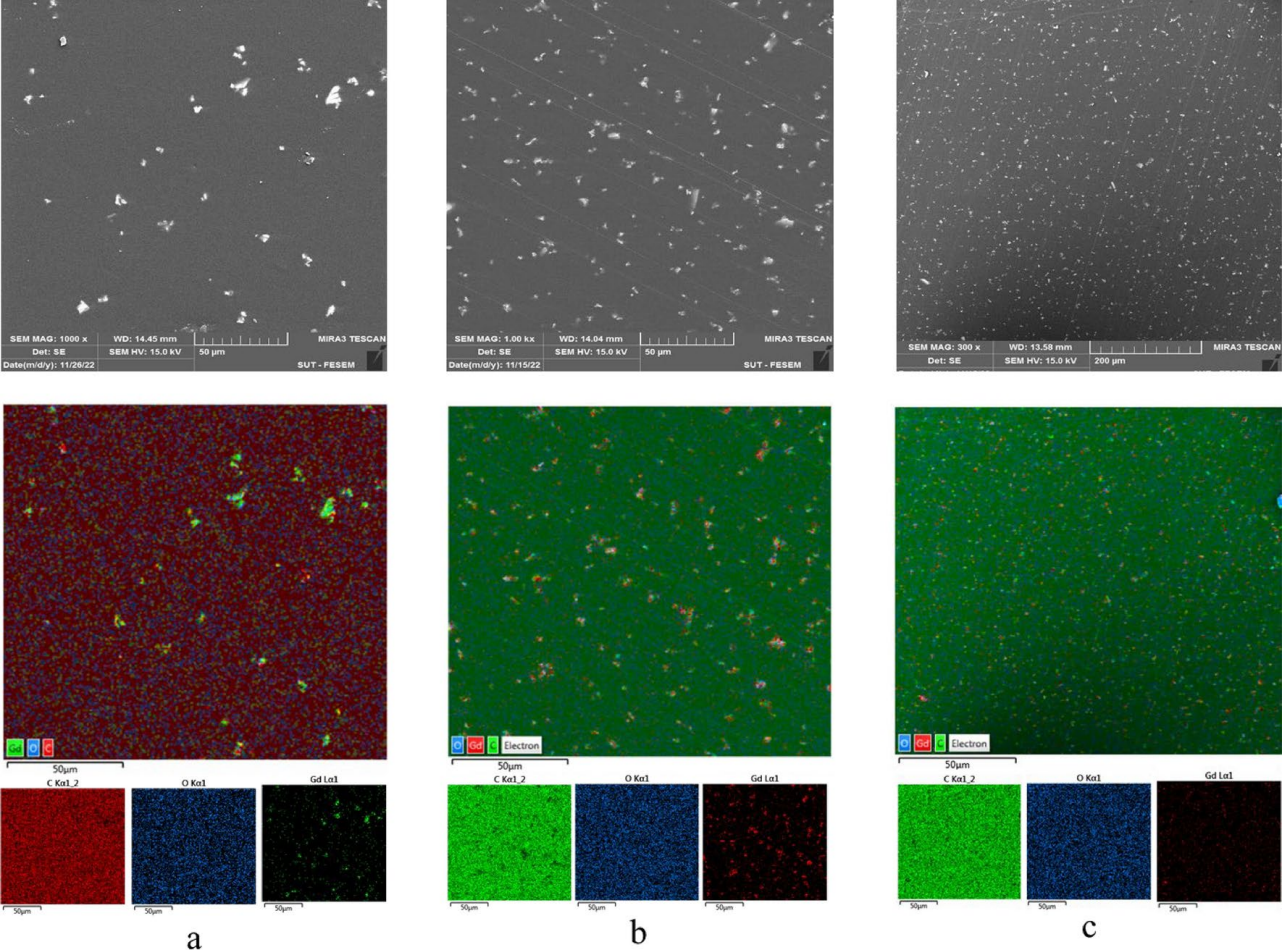
Elemental map of (**a**) 0.5%; (**b**) 2%; (**c**) 5% filler.

**Fig. 4:**
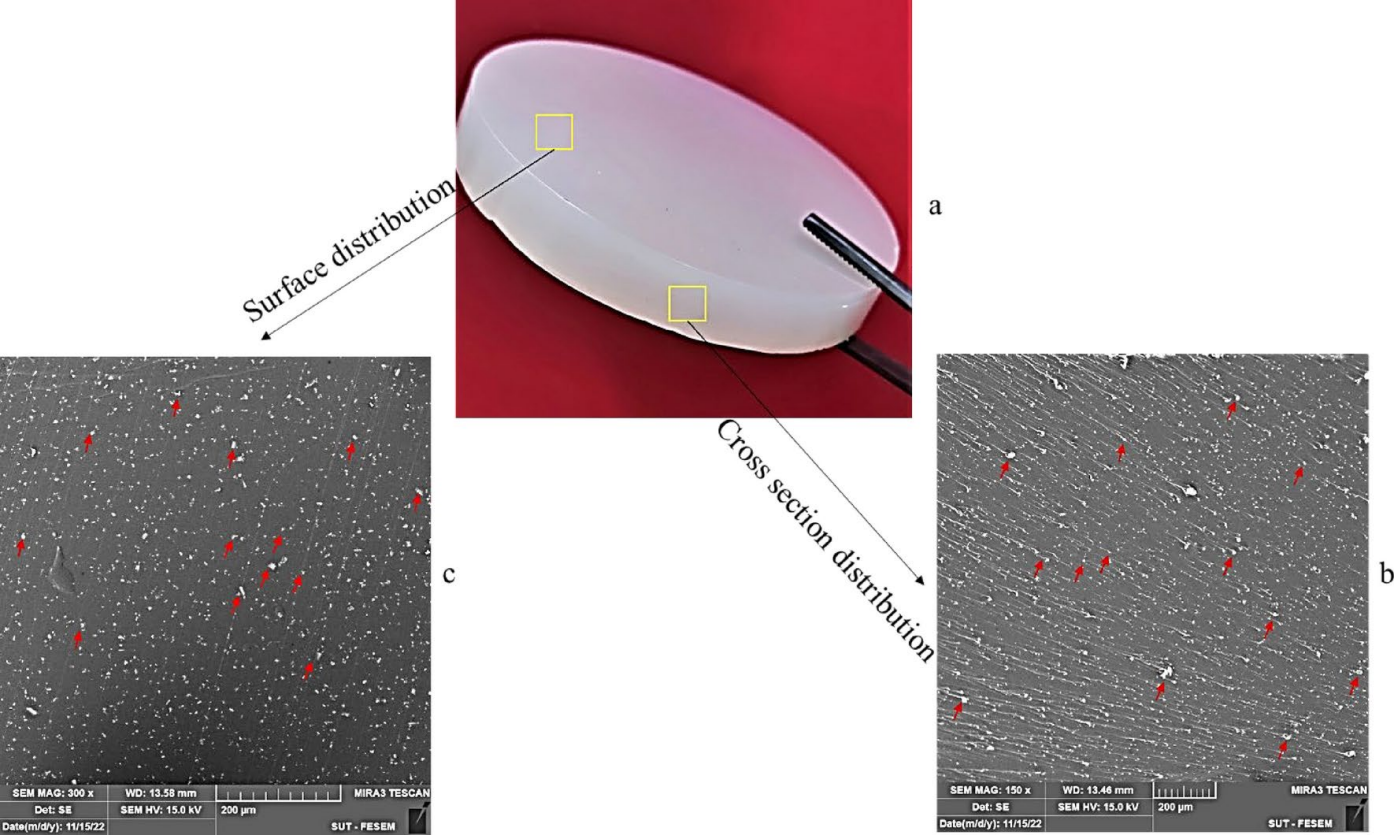
(**a**) epoxy/Gd_2_O_3_ composite. Distribution of Gd_2_O_3_ particles; (**b**) in the cross-section; (**c**) on the surface of the polymer.

Figure 3 demonstrates the elemental map of gadolinium within the composite, indicated by the green color dots. The absorber particles were dispersed in the polymeric matrix in an entirely random manner. Such homogeneous dispersion of particles was observed at different concentrations of Gd_2_O_3_. The neutron shield composite, including epoxy and dispersed Gd_2_O_3_, is demonstrated in Fig. 4a. As precipitation of denser gadolinium oxide might occur during the manufacturing of the composite, it was necessary to check if the Gd_2_O_3_ particles were homogeneously dispersed in the cured samples. For this purpose, we took the SEM images of the surface (Fig. 4b) and cross-section of the broken composite (Fig. 4c). The results demonstrated a uniform distribution of particles throughout the body of the samples.

**Fig. 5:**
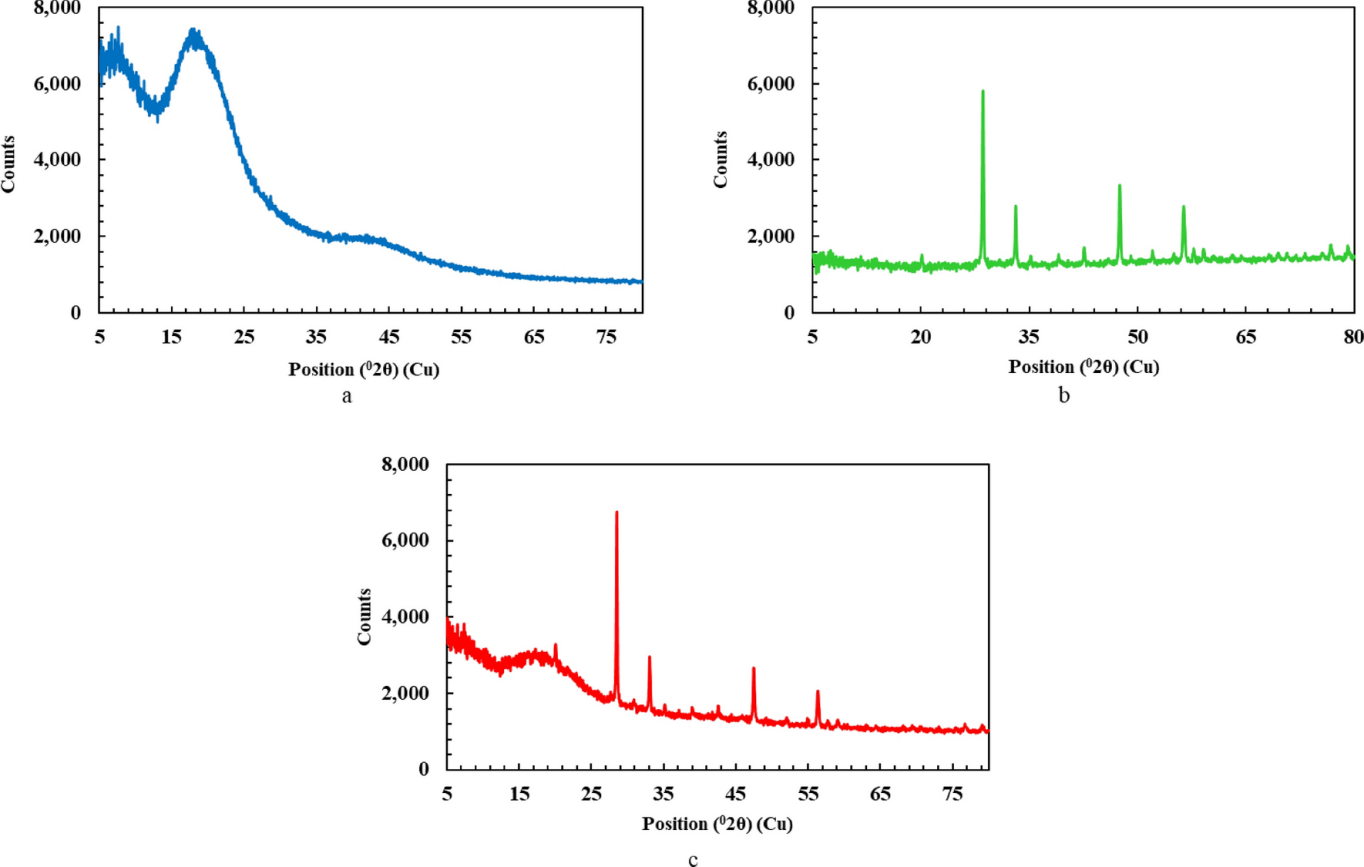
XRD spectrum of (**a**) epoxy, (**b**) Gd_2_O_3_, (**c**) epoxy/Gd_2_O_3_ composite.

Figure 5 displays the XRD spectra of epoxy (Fig. 5a), gadolinium oxide (Fig. 5b), and epoxy/gadolinium oxide composite (Fig. 5c). The peaks at $2\theta$ = 20.1, 28.5, 33.1, 35.5, 39.5, 42.6, 47.5, and 56.4 degrees are attributed to the prominent peaks of gadolinium oxide^31,34^. The broad peaks at $2\theta$ about 18 and a weak peak at 28 degrees are related to the epoxy polymer^35–37^. According to Fig. 5c, the formation of the composite had no significant effect on the crystalline structure of its Gd_2_O_3_ and epoxy constituents.

To study the molecular structure of the neat epoxy resin and the potential effect of composite formation on the functional groups of the polymer, samples of epoxy and epoxy/gadolinium oxide composite were analyzed by the FTIR ATR method.

**Fig. 6:**
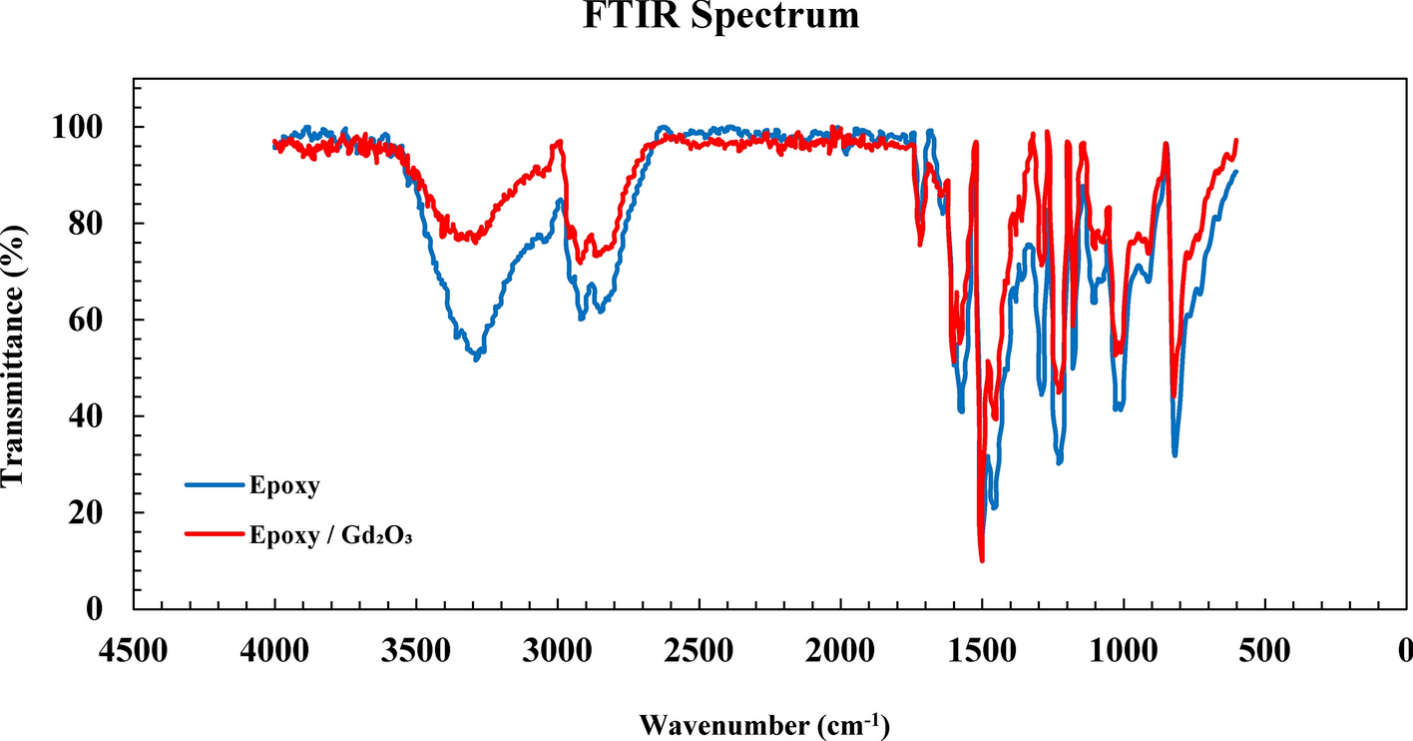
FTIR ATR spectrum of epoxy/Gd_2_O_3_ and epoxy.

In both spectra, the peak around 3200 cm^−1^ corresponds to the stretching vibration of O–H bond, the regions between 2800 and 2950 cm^−1^ are attributed to symmetric and asymmetric stretching of C–H bonds in CH_2_ and CH_3_ groups, the peak at 1720 cm^−1^ is related to the stretching vibration of carbonyl group C=O, and the observed peaks at 1630 and 1504 cm^−1^ refers to the stretching of C–C and C=C bonds in aromatic compounds. Also, the absorptions in the regions of 1288, 1220, and 1105 cm^−1^ are respectively ascribed to the stretching vibrations of C–O–C, C–O, and C–OH bonds. According to Fig. 6, the functional groups of the epoxy have not undergone significant changes due to the presence of Gd_2_O_3_ particles, so the addition of gadolinium oxide did not affect the molecular structure of the polymer, an observation that is in agreement with the previous studies^38,39^. In the ATR analysis, the radiation used has a limited penetration depth in the sample. When a composite sample is present with a filler on its surface, the incident beam, at some points, might less effectively penetrate the surface, which in turn, might cause lower intensities of the composite peaks.

**Fig. 7:**
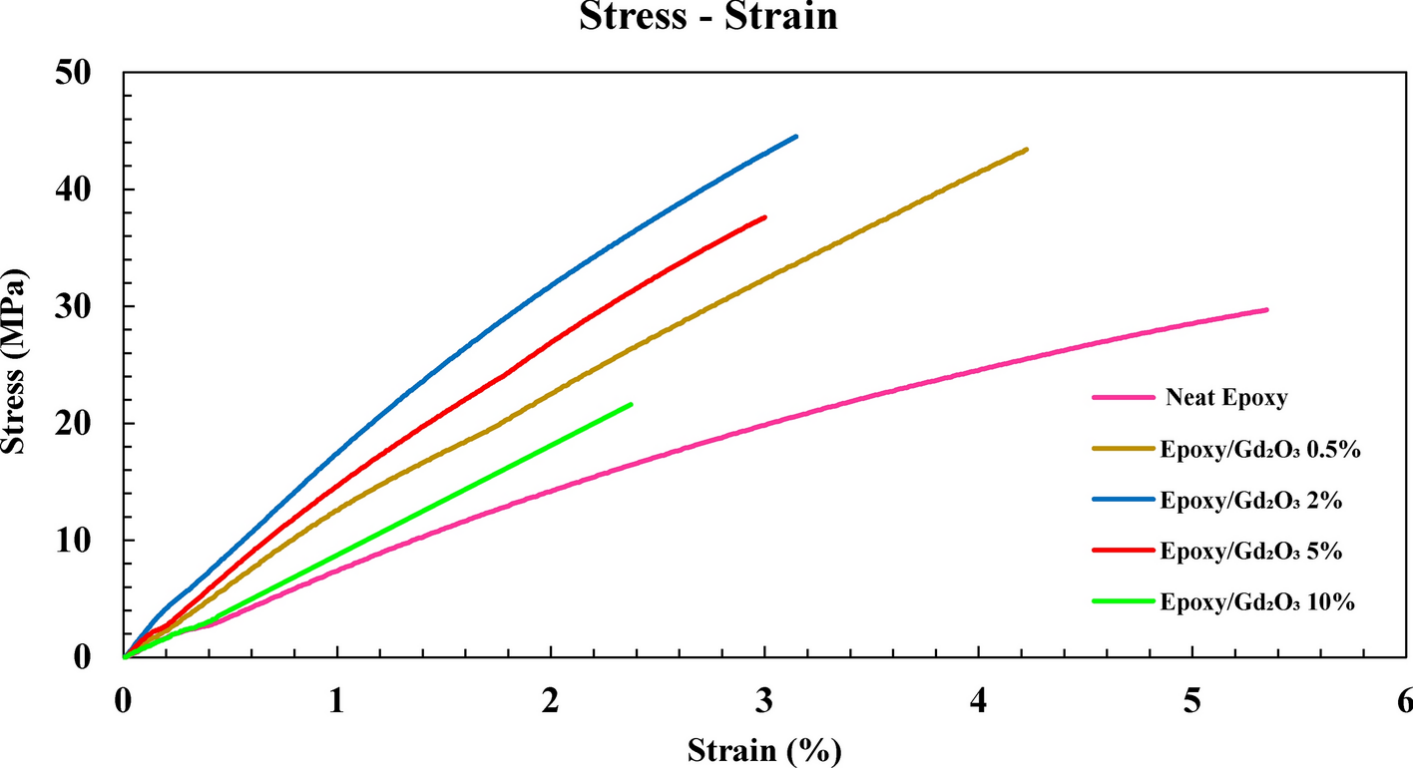
Stress–strain curve for neat epoxy and epoxy with different concentrations of Gd_2_O_3_.

**Fig. 8:**
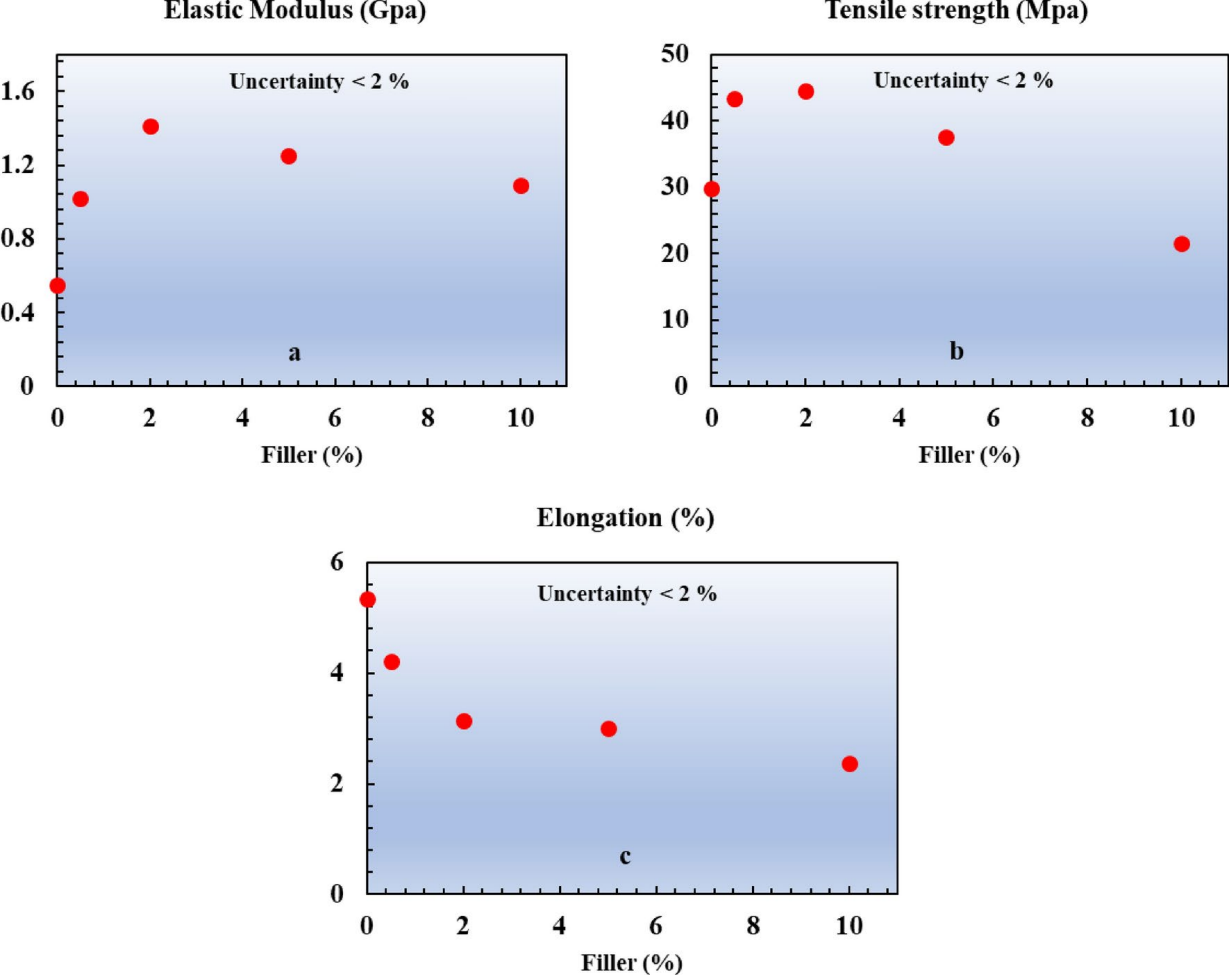
(**a**) Elastic modulus; (**b**) Tensile strength; (**c**) elongation of neat epoxy (0% filler) and other composites (0.5, 2, 5, 10% filler).

In the applications of the prepared composites, mechanical strength is often one of the most significant requirements. In order to evaluate this property, a tensile test was performed on the Gd_2_O_3_ -Epoxy composite. According to ISO 527 standard, three dumbbell-shaped specimens were made from the foregoing composite, a tensile test was carried out on all of the specimens, and the average value of strengths was used in the calculations. The test was repeated on the composites with different Gd_2_O_3_ concentrations. The gauge length has been considered to be 75 mm according to the ISO tensile standard. Initially, the pure epoxy sample was tested as the control sample, and the other samples were measured relative to it. The stress–strain curves are exhibited in Fig. 7. Also, the calculated values of the elastic modulus, tensile strength, and percentage of elongation at break are displayed in Fig. 8 for comparison.

**Fig. 9:**
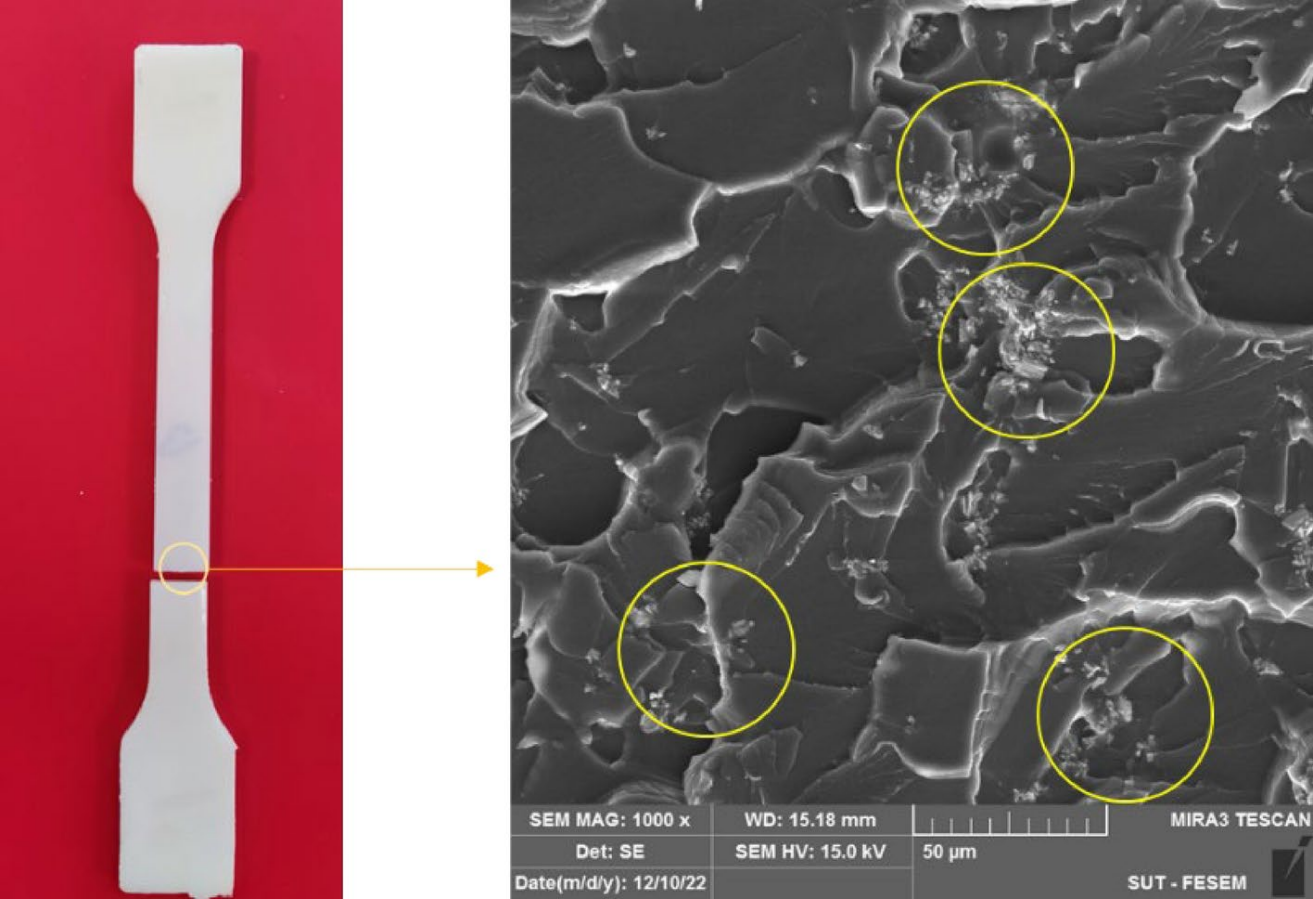
Cross-section SEM image of tensile 10% sample.

In Fig. 7, the stress–strain curves of all composite samples change almost linearly up to the point of fracture. This trend indicates the rigid nature of the fabricated composites, which has also been observed in other studies, which compounded tantalum oxide^40^, bismuth oxide^41^, and gadolinium oxide^42^ with epoxy in the composites. Based on the comparison of the tensile characteristics of different samples (Fig. 8), it can be concluded that by increasing of the percentage of Gd_2_O_3_, at first, elastic modulus and tensile strength increase, but above around 2% Gd_2_O_3_ content, they start diminishing. Such behavior can be explained by the following mechanisms: (1) At lower concentration of Gd_2_O_3_, where the epoxy matrix is still homogenous, satisfactory adhesion of resin and Gd_2_O_3_, causes effective transferring of the mechanical load to the stiffer gadolinium oxide, thereby enhancing its mechanical strength, and (2) Higher Gd_2_O_3_ content, ruins the uniformity of the matrix and the obtained composite cannot act as an integrated and cohesive material^43,44^. Note that the non-uniform distribution of metallic oxides at higher Gd_2_O_3_ concentrations may lead to the accumulation of particles in some points of the matrix. Such an effect is evidenced in Fig. 9. The points marked with yellow circles are the location of the particle accumulation in the 10% Gd_2_O_3_ sample. Apparently, sample fracture begins from these agglomerations. Besides the aforementioned effects, the percentage of elongation at the breakpoint showed a decreasing trend with the increasing Gd_2_O_3_ content.

The results of Fig. 8 are comparable to those reported by Prabhu et al. for the Ta2O5-reinforced composite^40^. Based on the results of the mechanical tests of the current study, the optimal concentration of Gd_2_O_3_ in the epoxy composite was found to be around 2% among our samples.

Thermogravimetric Analysis (TGA) was performed on the plain and Gd_2_O_3_-containing composites to scrutinize their thermal resistances and the potential changes imposed on Gd_2_O_3_-containing samples during the fabrication. Our TG tests were started at the ambient temperature and reached to700 °C, with a 10 $\frac{{^\circ {\text{C}}}}{\min }$ growing rate, in the nitrogen atmosphere.

**Fig. 10:**
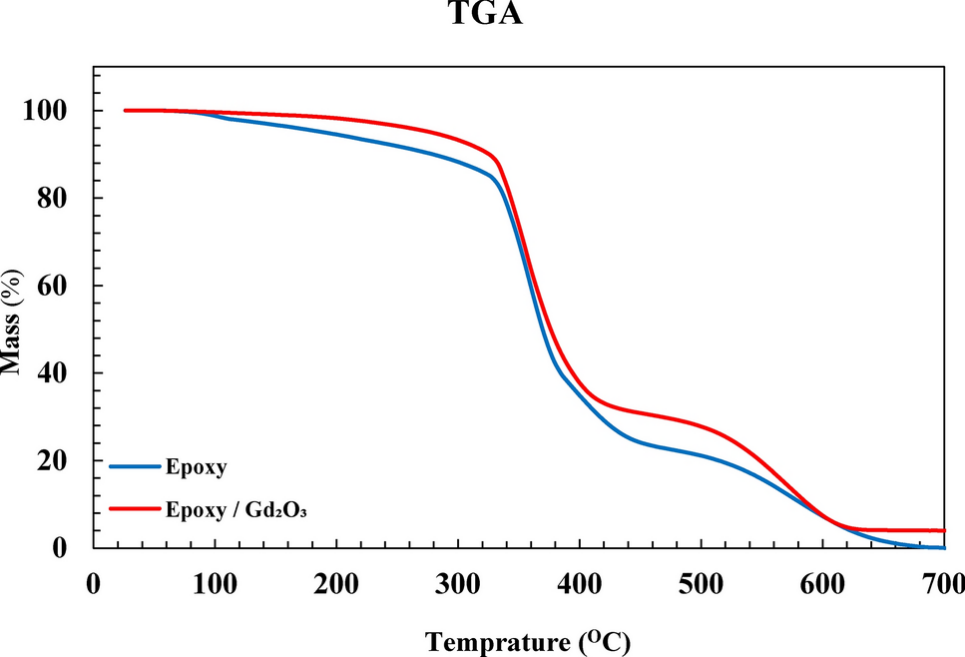
TG curves of neat epoxy and composite.

According to Fig. 10, the highest percentage of the degradation occurred at 350 °C, but samples were completely decomposed at 700 °C. Adding the gadolinium oxide to the polymeric matrix had a positive effect on the thermal stability of the composite, likely because compounding with Gd_2_O_3_ increases the thermal conductivity of the samples, which in turn leads to a better dissipation of destructive thermal energy from the composite specimens. Similar results were reported by the previous researchers^45–47^.

### Neutron shielding performance

**Fig. 11:**
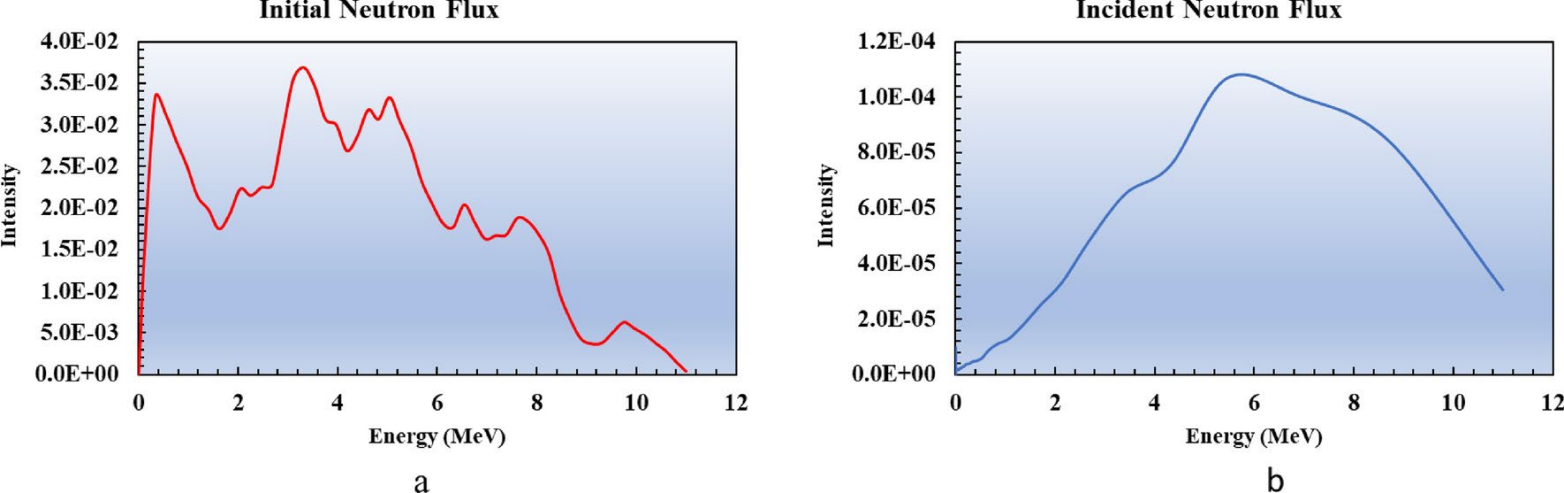
Initial (**a**) and incident (**b**) neutron flux.

Epoxy samples with 0.5, 2, 5, and 10% Gd_2_O_3_ content underwent neutron attenuation experiments. For each of the aforementioned mass percentages, the neutronic test was carried out on the specimens with thicknesses ranging from 1 to 4 cm. All experiments and simulations were repeated three times, and their average values were reported. Neutron shielding simulation was carried out using the Monte Carlo particle transport model using the MCNPX code. At the large sample thicknesses, neutron moderation is significant, and scattering and absorption cross-sections increase as a consequence. This change in the energy of the neutron beam leads to a greater effect of changes in the gadolinium mass fraction in thicker samples than in thinner ones. Note that any reduction in the neutron energy increases the absorption rate much more than the scattering rate at the fast and epithermal energies. The initial neutron flux, comparable to Am-Be neutron flux^48^, and incident neutron flux, calculated by the MCNP simulation, are displayed in Fig. 11a and b, respectively, and a decrease in neutron flux can be observed.

**Fig. 12:**
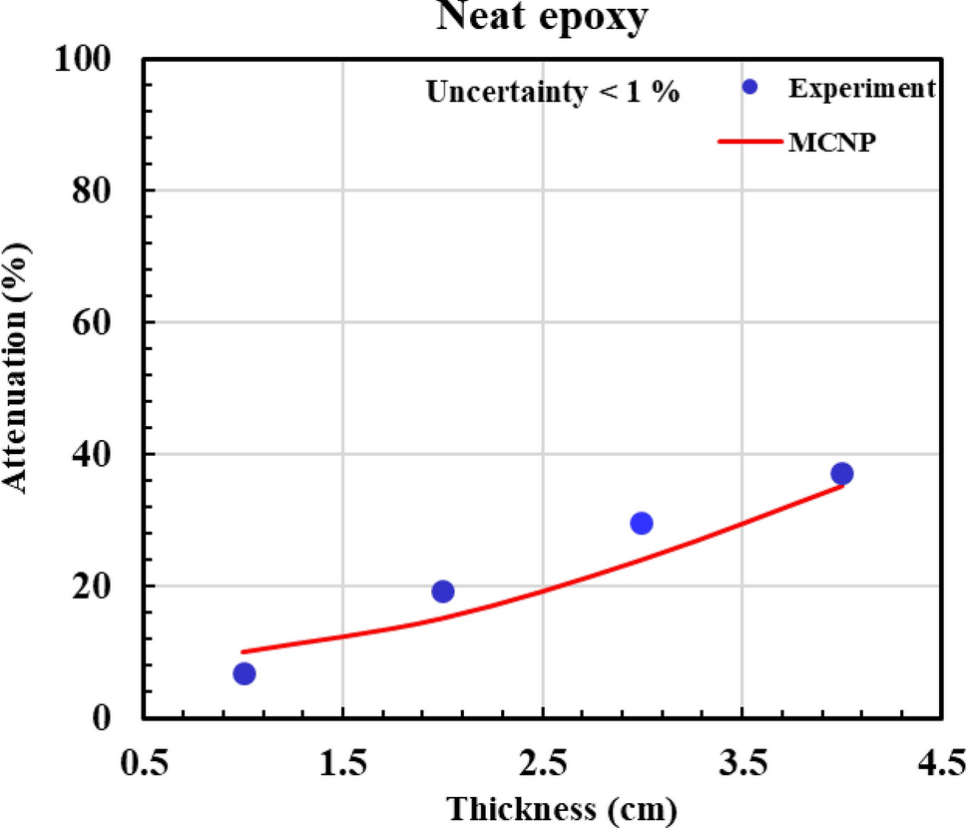
Experimental and simulation neutron attenuation of neat epoxy.

**Fig. 13:**
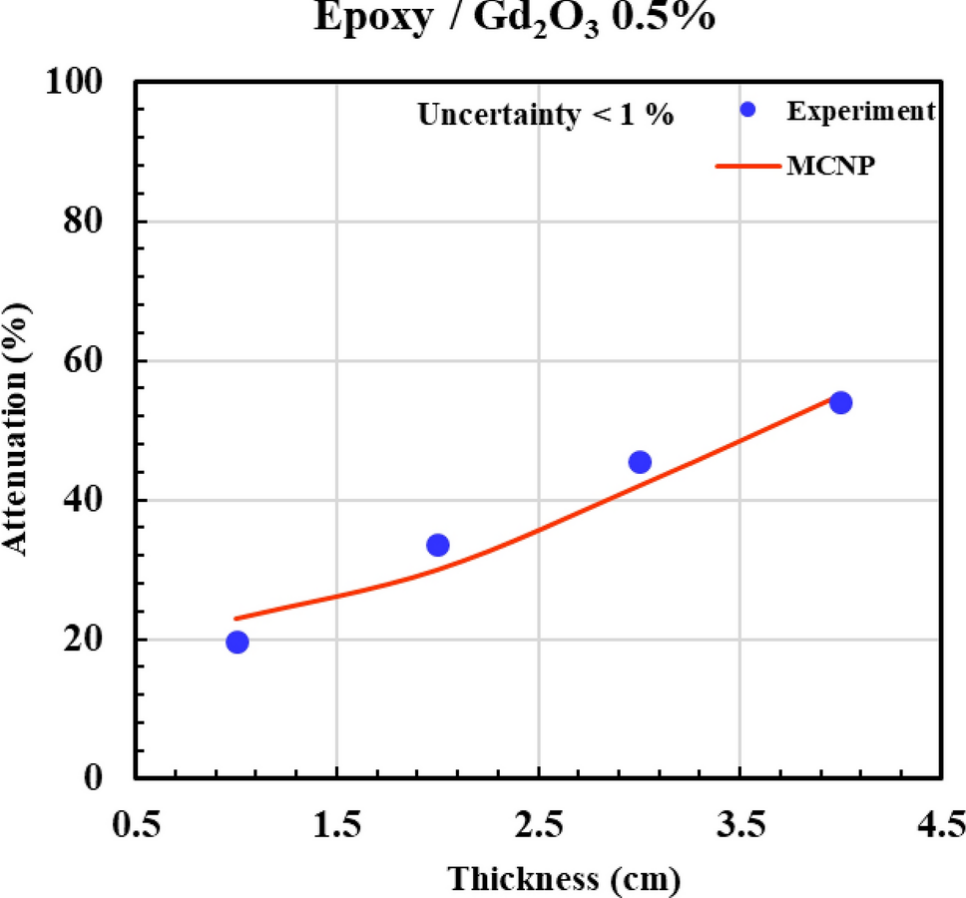
Experimental and simulation neutron attenuation of epoxy/Gd_2_O_3_ 0.5%.

**Fig. 14:**
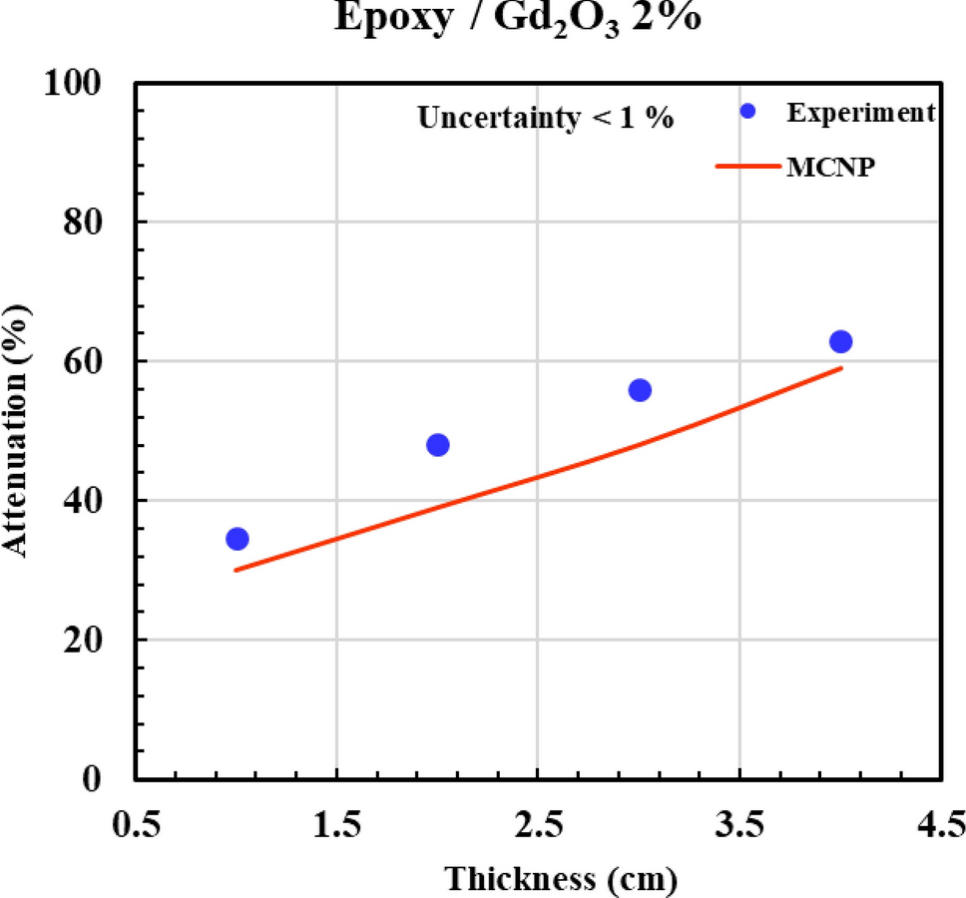
Experimental and simulation neutron attenuation of epoxy/Gd_2_O_3_ 2%.

**Fig. 15:**
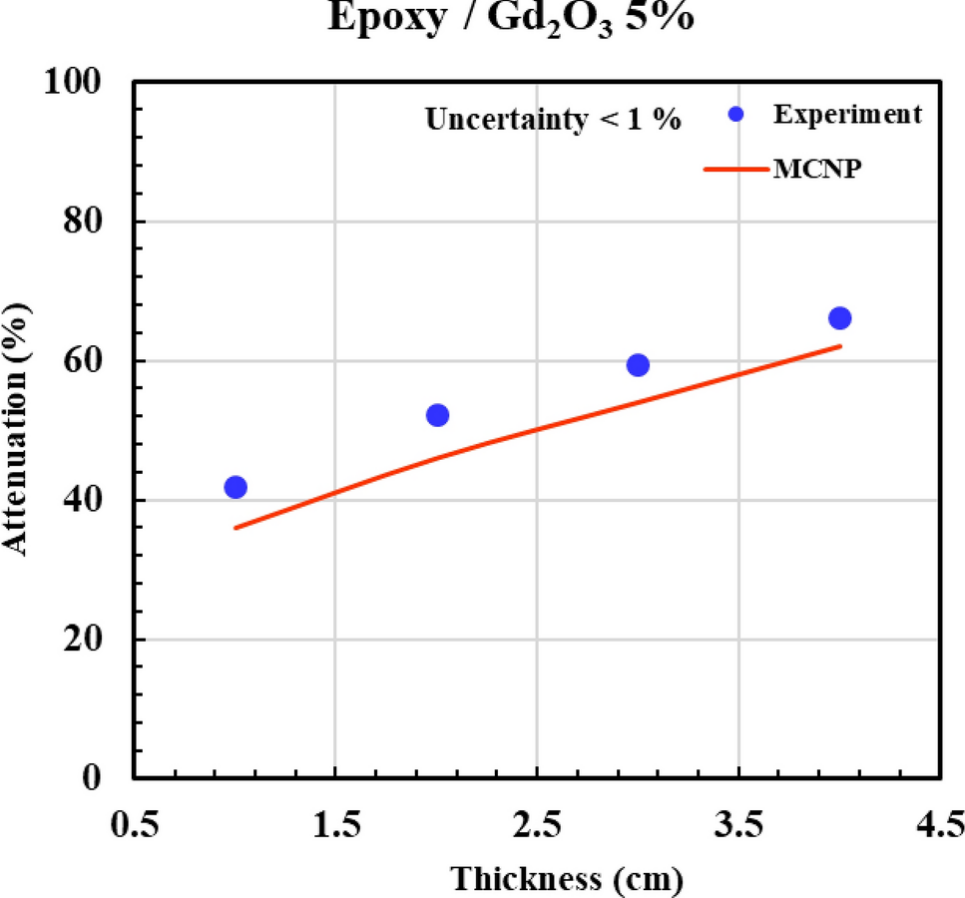
Experimental and simulation neutron attenuation of epoxy/Gd_2_O_3_ 5%.

**Fig. 16:**
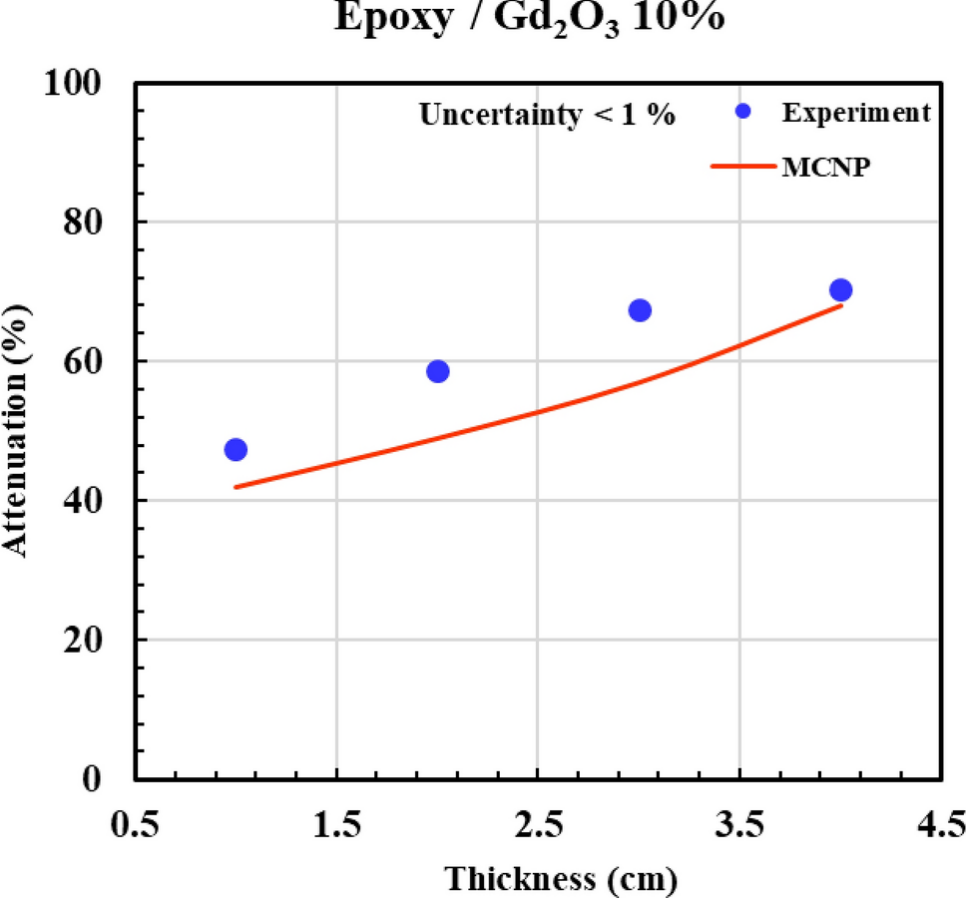
Experimental and simulation neutron attenuation of epoxy/Gd_2_O_3_ 10%.

According to Figs. 12, 13, 14, 15 and 16 and Table 3, all composites with 0.5%, 2%, 5%, and 10% Gd_2_O_3_ content performed better than the neat epoxy in terms of neutron absorption, as we can see the thickest neat epoxy sample can absorb 37% of neutrons but by adding 0.5% Gd_2_O_3,_ the efficiency of the composite enhances significantly. Most notably, the composite with 10 wt% gadolinium oxide can absorb 70% of the incident neutrons at a thickness of 4 cm, demonstrating better performance than a 4 cm thickness of concrete, which can absorb only 25% of the neutrons^49^. The results indicate that Gd_2_O_3_-bearing composites are effective materials for neutron shielding, decreasing neutron dose. Here, there is a point that deserves attention: Although neutron capture increases with increasing both thickness and absorber content, absorption of the neutron in ^157^Gd isotope is accompanied by the production of gamma rays and characteristic X-rays, which, on the other hand, increase the total dose of the personnel. As a result, the optimum values of Gd_2_O_3_ and composite thickness are those values that minimize the total dose of “neutron + gamma” and simultaneously meet the economic considerations. Engaging in such optimization processes that require detailed information about the neutron reactions and cost analysis is beyond the scope of the current study. Nevertheless, a specific sample may be fabricated based on demand and environmental conditions.

**Table 3:** Attenuation factor through experiment and simulation.

Thickness (cm)	Attenuation factor (ΔI/I_0_ × 100)									
	0.0% Absorber		0.5% Absorber		2.0% Absorber		5.0% Absorber		10.0% Absorber	
	Exp (%)	Sim (%)	Exp (%)	Sim (%)	Exp (%)	Sim (%)	Exp (%)	Sim (%)	Exp (%)	Sim (%)
1	7	10	20	23	34	30	42	36	47	42
2	19	16	34	30	48	39	52	46	58	49
3	30	24	46	42	56	48	59	54	66	57
4	37	35	54	55	63	59	66	62	70	68
Average absolute error (AAE, %)									12.8	

Table 3 presents the experimental and computational values of the attenuation factor (Af) for the different examined samples. The last row of the table indicates an average absolute error (*AAE*, %) that is defined as:

6 $$AAE,\% = \frac{{\sum\limits_{1}^{n} {\frac{{\left| {Af_{cal.} - Af_{\exp .} } \right|}}{{Af_{\exp .} }}} }}{n} \times 100$$

where indices “cal.” and “exp.” denote the calculated and experimental values of “*Af* “, respectively, and “n” refers to the total number of the experiment. According to Table 3, (*AAE* %) is about 12.5%, which shows a relatively fair agreement between simulation and experiment. The discrepancy between the experimental and simulation results can be attributed to unavoidable experimental errors, environmental factors, and variations in materials and their impurities.

In addition to the attenuation factor (*Af* ), the experimental data and calculations resulted in the effective macroscopic absorption cross-section (Σ_*a*_), which can be determined from the following equation:

7 $$I = I_{0} e^{{ - \Sigma_{a} x}}$$

where $\Sigma_{a}$ is the absorption macroscopic cross-section, $x$ is shield thickness, $I$ and $I_{0}$ are the number of neutrons in the incident and passed beam that either was detected experimentally or generated by simulation.

The data presented in Table 4 indicate that the macroscopic absorption cross-section increases nonlinearly with the Gd_2_O_3_ content. This observation could be attributed to the increased likelihood of microbubble and porosity formation when higher filler concentrations are used. Additionally, the formation of agglomerates becomes more prevalent at higher concentrations, which may have a detrimental effect on the shielding performance. The average error of MCNP in the simulation of the experiments was about 14%, which is fairly acceptable (Table 4).

**Table 4:** Macroscopic absorption cross-section, based on experiment and simulation.

Macroscopic absorption cross section (Σ_*a*_, cm^-1^)		
Gd_2_O_3_ (w/w, %)	Experiment	MCNP
0.5	0.242	0.261
2	0.425	0.356
5	0.541	0.446
10	0.643	0.544
Average absolute error (AAE, %)		14.26

Self-shielding is a phenomenon in which the absorbing material (e.g., Gd_2_O_3_) on the surface of a shield excessively absorbs the neutron radiation and reduces the flux that reaches the internal layers. As a result, the inner parts of the shield that receive diminished neutron flux cannot effectively contribute to absorption, and ultimately, the overall performance of the shield decreases. To be more accurate in our calculations, we examined the possibility of such a phenomenon occurring. Our analyses revealed that the mean free path of neutrons (λ), as derived from Eq. 8, is greater than the thickness of the shield, and self-shielding can be we disregarded.

8 $$\lambda = \frac{1}{\sum }$$

## Conclusion

A new neutron shielding composite was fabricated through homogeneous dispersion of gadolinium oxide particles in epoxy resin. According to the results of XRD and FTIR analyses, both components of the composite preserved their identities, and no significant chemical interactions were observed between them. SEM images indicated that Gd_2_O_3_ particles were well distributed throughout the body and surface of the composites, in a random fashion. The TGA analysis revealed that the addition of Gd_2_O_3_ had a positive effect on the thermal resistance of the epoxy matrix. The new epoxy/Gd_2_O_3_ displayed an acceptable mechanical strength (e.g., the sample encompassing 2% Gd_2_O_3_ had a strength of 44.5 MPa with an elongation rate of 3.1%.

The neutron shielding performance of the fabricated composite was evaluated by both experiments and simulations. The results demonstrated that the new composite offers appreciable neutron absorption performance, with a 70% reduction in neutron flux when 10% Gd_2_O_3_ was added into the epoxy polymer. The accuracy of the MCNP code in simulating the neutronic data was notable, as it could compute the attenuation factor and macroscopic absorption cross-section, with average errors of 12.8% and 14.26%, respectively. Due to the large mean free path of neutrons in the samples, the self-shielding effect was ignorable.

The new neutron shield exhibits several advantages over traditional shields, such as concrete. These advantages include low density, cost-effectiveness, high mechanical strength, and convenient transportation. As a result, the new shield can be utilized in a wide range of applications, including a protective neutron shield, which plays a crucial role in designing treatment rooms for cancer radiotherapy by ensuring patient safety from harmful radiation. Additionally, the new composite material can be incorporated into the structure of linear accelerators, acting as a pivotal component to filter out unwanted neutron radiation, thereby enhancing the safety of medical equipment used in radiation therapy^23,50^. Applying the fabricated composite in the manufacture of the dry casks for the interim storgae of spent nuclear fuels is of particular significance. To elaborate on this, we must go through some details, such as: When spent nuclear fuels are discharged from the reactors, they are stored in the spent nuclear pool, where they are cooled by the appropriate heat transfer equipment and lose the bulk of their initial radioactivity. When the level of activity decreases under a certain standard level and fuels no longer generate considerable decay heat, they are transferred to the dry casks to be stored for interim periods (up to tens of years). In such capacity, the dry cask must comprise a neutron shield layer to protect the personnel from spontaneous-fission neutrons of the transuranium elements, and our Gd_2_O_3_-epoxy composite very well fits with the necessary requirements of such a cask.

Overall, the novelty of this study is the development of a lightweight, high-efficiency neutron shield that can be substituted with heavier shields, like concrete, in applications that are important in both industry and medicine.

## Data Availability

The corresponding author holds the experimental datasets and simulation source codes, which can be provided upon reasonable request.

## References

[1] Tyagi, G., Singhal, A., Routroy, S., Bhunia, D. & Lahoti, M. Radiation shielding concrete with alternate constituents: An approach to address multiple hazards. *J. Hazard. Mater.* **404**, 124201. 10.1016/j.jhazmat.2020.124201 (2021). 33129018 10.1016/j.jhazmat.2020.124201

[2] Pomaro, B. A review on radiation damage in concrete for nuclear facilities: From experiments to modeling. *Model. Simul. Eng.* **2016**, 4165746. 10.1155/2016/4165746 (2016).

[3] Lakshminarayana, G. et al. Investigation of structural, thermal properties and shielding parameters for multicomponent borate glasses for gamma and neutron radiation shielding applications. *J. Non-Cryst. Solids* **471**, 222–237. 10.1016/j.jnoncrysol.2017.06.001 (2017).

[4] Sayyed, M. I. Investigation of shielding parameters for smart polymers. *Chin. J. Phys.* **54**, 408–415. 10.1016/j.cjph.2016.05.002 (2016).

[5] Winkler, B. Applications of neutron radiography and neutron tomography. *Rev. Mineral. Geochem.* **63**, 459–471. 10.2138/rmg.2006.63.17 (2006).

[6] Kardjilov, N. et al. Industrial applications at the new cold neutron radiography and tomography facility of the HMI. *Nucl. Instrum. Methods Phys. Res., Sect. A* **542**, 16–21. 10.1016/j.nima.2005.01.005 (2005).

[7] Ahmed, Y. A., Balogun, G. I., Jonah, S. A. & Funtua, I. I. The behavior of reactor power and flux resulting from changes in core-coolant temperature for a miniature neutron source reactor. *Ann. Nucl. Energy* **35**, 2417–2419. 10.1016/j.anucene.2008.08.005 (2008).

[8] Ardiansyah, A. et al. Science mapping for concrete composites as radiation shielding: A review. *Radiat. Phys. Chem.* **207**, 110835. 10.1016/j.radphyschem.2023.110835 (2023).

[9] Trtik, P. et al. Improving the spatial resolution of neutron imaging at paul scherrer institut–the neutron microscope project. *Phys. Procedia* **69**, 169–176 (2015).

[10] Nabil, I. M., El-Samrah, M. G., Omar, A., Tawfic, A. F. & El Sayed, A. F. Experimental, analytical, and simulation studies of modified concrete mix for radiation shielding in a mixed radiation field. *Sci. Rep.* **13**, 17637. 10.1038/s41598-023-44978-8 (2023). 37848620 10.1038/s41598-023-44978-8 PMC10582154

[11] Ali, M. A. E. M., Tawfic, A. F., Abdelgawad, M. A., Mahdy, M. & Omar, A. Gamma and neutrons shielding using innovative fiber reinforced concrete. *Prog. Nucl. Energy* **145**, 104133. 10.1016/j.pnucene.2022.104133 (2022).

[12] Yadollahi, A., Nazemi, E., Zolfaghari, A. & Ajorloo, A. M. Optimization of thermal neutron shield concrete mixture using artificial neural network. *Nucl. Eng. Des.* **305**, 146–155. 10.1016/j.nucengdes.2016.05.012 (2016).

[13] Oğul, H. et al. Gamma and neutron shielding parameters of polyester-based composites reinforced with boron and tin nanopowders. *Radiat. Phys. Chem.* **201**, 110474. 10.1016/j.radphyschem.2022.110474 (2022).

[14] Akhdar, H. Theoretical investigation of fast neutron and gamma radiation properties of polycarbonate-bismuth oxide composites using Geant4. *Nanomaterials* **12**, 3577 (2022). 36296770 10.3390/nano12203577 PMC9609593

[15] Bîrcă, A., Gherasim, O., Grumezescu, V. & Grumezescu, A. M. *Materials for biomedical engineering* 1–28 (Elsevier, Amsterdam, 2019).

[16] Kavetskiy, A., Yakubova, G., Sargsyan, N., Prior, S. A. & Torbert, H. A. (2023) *Encyclopedia of Soils in the Environment (Second Edition)* In: Michael J. Goss & Margaret Oliver (eds.). Academic Press. Pp. 625–641

[17] Rusov, V. D., Tarasov, V. A., Chernezhenko, S. A., Kakaev, A. A. & Smolyar, V. P. Neutron moderation theory with thermal motion of the moderator nuclei. *Eur. Phys. J. A* **53**, 179. 10.1140/epja/i2017-12363-9 (2017).

[18] Morris, A. S. & Langari, R. (2021) in *Measurement and Instrumentation (Third Edition)* In: (eds.) Alan S. Morris & Reza Langari, Academic Press, pp. 637–677.

[19] Sharma, B. P. (2001) in *Encyclopedia of Materials: Science and Technology.* In: K. H. Jürgen Buschow *et al. *pp. 6365–6369 Elsevier, UK

[20] Nambiar, S. & Yeow, J. T. W. Polymer-composite materials for radiation protection. *ACS Appl. Mater. Interfaces* **4**, 5717–5726. 10.1021/am300783d (2012). 23009182 10.1021/am300783d

[21] Shang, Y. et al. Multilayer polyethylene/hexagonal boron nitride composites showing high neutron shielding efficiency and thermal conductivity. *Compos. Commun.* **19**, 147–153. 10.1016/j.coco.2020.03.007 (2020).

[22] Vegari, A., Abdisaray, A., Mostafanejad, K. & Jabbari, N. High-density polyethylene (HDPE)-incorporated boron carbide and boric acid nanoparticles as a nanoshield of photoneutrons from medical linear accelerators. *Int. J. Radiat. Biol.* **100**, 609–618 (2024). 38190436 10.1080/09553002.2023.2295964

[23] Hassanpour, M., Faruque, M. R. I., Hassanpour, M., Khandaker, M. U. & Al-mugren, K. S. Evaluation of the impact of PL/hBN and PL/B_4_C on the neutron leakage generated by the LINAC in the treatment room. *Alex. Eng. J.* **105**, 98–104. 10.1016/j.aej.2024.06.084 (2024).

[24] Ghassoun, J. & Senhou, N. The evaluation of neutron and gamma ray dose equivalent distributions in patients and the effectiveness of shield materials for high energy photons radiotherapy facilities. *Appl. Radiat. Isot.* **70**, 620–624. 10.1016/j.apradiso.2011.12.041 (2012). 22257567 10.1016/j.apradiso.2011.12.041

[25] Wang, B. et al. Properties and thermal neutron areal transmittance of a B_4_C filled thermoplastic elastomer based rubber composite. *Nucl. Mater. Energy* **31**, 101193. 10.1016/j.nme.2022.101193 (2022).

[26] Okuno, K. Neutron shielding material based on colemanite and epoxy resin. *Radiat. Prot. Dosim.* **115**, 258–261. 10.1093/rpd/nci154 (2005). 10.1093/rpd/nci154 16381724

[27] Stacey, W. M. *Nuclear reactor physics* (John Wiley & Sons, New York, 2018).

[28] Adeli, R., Shirmardi, S. P. & Ahmadi, S. J. Neutron irradiation tests on B_4_C/epoxy composite for neutron shielding application and the parameters assay. *Radiat. Phys. Chem.* **127**, 140–146. 10.1016/j.radphyschem.2016.06.026 (2016).

[29] Das, A., Ray, A. & Singh, T. Tungsten-based polymer composite, a new lead-free material for efficient shielding of coupled neutron-gamma radiation fields: A FLUKA simulation study. *Phys. Scripta* **98**, 115302. 10.1088/1402-4896/acfa3e (2023).

[30] Kiani, M. A., Ahmadi, S. J., Outokesh, M., Adeli, R. & Mohammadi, A. Preparation and characteristics of epoxy/clay/B_4_C nanocomposite at high concentration of boron carbide for neutron shielding application. *Radiat. Phys. Chem.* **141**, 223–228. 10.1016/j.radphyschem.2017.07.013 (2017).

[31] İrim, ŞG. et al. Physical, mechanical and neutron shielding properties of h-BN/Gd_2_O_3_/HDPE ternary nanocomposites. *Radiat. Phys. Chem.* **144**, 434–443. 10.1016/j.radphyschem.2017.10.007 (2018).

[32] Castley, D., Goodwin, C. & Liu, J. Computational and experimental comparison of boron carbide, gadolinium oxide, samarium oxide, and graphene platelets as additives for a neutron shield. *Radiat. Phys. Chem.* **165**, 108435. 10.1016/j.radphyschem.2019.108435 (2019).

[33] Toyen, D., Wimolmala, E., Sombatsompop, N., Markpin, T. & Saenboonruang, K. Sm2O3/UHMWPE composites for radiation shielding applications: Mechanical and dielectric properties under gamma irradiation and thermal neutron shielding. *Radiat. Phys. Chem.* **164**, 108366. 10.1016/j.radphyschem.2019.108366 (2019).

[34] Khan, S. A., Gambhir, S. & Ahmad, A. Extracellular biosynthesis of gadolinium oxide (Gd_2_O_3_) nanoparticles, their biodistribution and bioconjugation with the chemically modified anticancer drug taxol. *Beilstein J. Nanotechnol.* **5**, 249–257 (2014). 24778946 10.3762/bjnano.5.27 PMC3999844

[35] Ho, M.-W., Lam, C.-K., Lau, K.-T., Ng, D. H. L. & Hui, D. Mechanical properties of epoxy-based composites using nanoclays. *Compos. Struct.* **75**, 415–421. 10.1016/j.compstruct.2006.04.051 (2006).

[36] Abdullah, S. I. & Ansari, M. N. M. Mechanical properties of graphene oxide (GO)/epoxy composites. *HBRC Journal* **11**, 151–156. 10.1016/j.hbrcj.2014.06.001 (2015).

[37] Parameswaranpillai, J., Pulikkalparambil, H., Rangappa, S. M. & Siengchin, S. *Epoxy Composites* (Wiley Online Library, New York, 2021).

[38] Sahmetlioglu, E., Mart, H., Yuruk, H. & Sürme, Y. Synthesis and characterization of oligosalicylaldehyde-based epoxy resins. *Chem. Pap.-Slovak Acad. Sci.* **60**, 65–68. 10.2478/s11696-006-0012-1 (2006).

[39] Duraibabu, D., Alagar, M. & Kumar, S. A. Studies on mechanical, thermal and dynamic mechanical properties of functionalized nanoalumina reinforced sulphone ether linked tetraglycidyl epoxy nanocomposites. *RSC Adv.* **4**, 40132–40140 (2014).

[40] Prabhu, S., Bubbly, S. G. & Gudennavar, S. B. Thermal, mechanical and γ-ray shielding properties of micro- and nano-Ta_2_O_5_ loaded DGEBA epoxy resin composites. *J. Appl. Polym. Sci.* **138**, 51289. 10.1002/app.51289 (2021).

[41] Muthamma, M. V., Prabhu, S., Bubbly, S. G. & Gudennavar, S. B. Micro and nano Bi_2_O_3_ filled epoxy composites: Thermal, mechanical and γ-ray attenuation properties. *Appl. Radiat. Isot.* **174**, 109780. 10.1016/j.apradiso.2021.109780 (2021). 34052516 10.1016/j.apradiso.2021.109780

[42] Li, R. et al. Effect of particle size on gamma radiation shielding property of gadolinium oxide dispersed epoxy resin matrix composite. *Mater. Res. Express* **4**, 035035 (2017).

[43] Husseinsyah, S. & Ahmad, R. Properties of low-density polyethylene/palm kernel shell composites: Effect of polyethylene co-acrylic acid. *J. Thermoplast. Compos. Mater.* **26**, 3–15. 10.1177/0892705711417028 (2013).

[44] Kiran, M., Govindaraju, H., Jayaraju, T. & Kumar, N. Effect of fillers on mechanical properties of polymer matrix composites. *Mater. Today: Proc.* **5**, 22421–22424 (2018).

[45] Sun, J. et al. Lignin epoxy composites: Preparation, morphology, and mechanical properties. *Macromol. Mater. Eng.* **301**, 328–336 (2016).

[46] Guo, Q. et al. One-pot synthesis of bimodal silica nanospheres and their effects on the rheological and thermal–mechanical properties of silica–epoxy composites. *RSC Adv.* **5**, 50073–50081. 10.1039/C5RA06914A (2015).

[47] Nazarenko, O., Melnikova, T. & Visakh, T. Thermal and mechanical characteristics of polymer composites based on epoxy resin, aluminium nanopowders and boric acid. *J. Phys.: Conf. Ser.* **671**, 012040. 10.1088/1742-6596/671/1/012040 (2016).

[48] Hatefi Moadab, N. & Kheradmand Saadi, M. Optimization of an Am-Be neutron source shield design by advanced materials using MCNP code. *Radiat. Phys. Chem.* **158**, 109–114. 10.1016/j.radphyschem.2019.01.026 (2019).

[49] Gallego, E., Lorente, A. & Vega-Carrillo, H. R. Testing of a high-density concrete as neutron shielding material. *Nucl. Technol.* **168**, 399–404. 10.13182/NT09-A9216 (2009).

[50] Jia, S. B., Soleimani, A., Mirsadraee, M., Zarifi, S. & Sanaeifar, E. Evaluation of the effectiveness of testicular shielding in rectal cancer radiotherapy. *Radiat. Phys. Chem.* **202**, 110435 (2023).

